# Adult Congenital Heart Disease Outpatient Clinic. Descriptive Analysis of A 12-Year Experience in Brazil

**DOI:** 10.21470/1678-9741-2019-0047

**Published:** 2020

**Authors:** Fernando Amaral, Paulo Henrique Manso, Maria Fernanda Balthazar Jacob, André Schmidt

**Affiliations:** 1 Cardiology Center of the Department of Internal Medicine, Faculdade de Medicina de Ribeirão Preto da Universidade de São Paulo (FMRP-USP), Ribeirão Preto, SP, Brazil.

**Keywords:** Heart Septal Defects, Ventricular, Aortic Coarctation, Tetralogy of Fallot, Heart Defects, Congenital, Aortic Valve Stenosis, Lost to Follow-up, Adult

## Abstract

**Objective:**

Description of adult congenital heart disease (CHD) outpatient characteristics has not been reported and several aspects regarding these patients require attention. We describe the 12-year experience of a Brazilian unit.

**Methods:**

The main characteristics of 1168 patients were reviewed annotating for each patient age, gender, city of residence, main diagnosis, functional class at last examination, defect complexity and in-hospital referral pattern.

**Results:**

Increasing workload was documented. Among the CHD patients, 663 (57%) were between 14 and 30 years old and 920 (79%) lived in the referral region. Referrals were made by hospital cardiologists for 611 (52%) patients, while 519 (45%) were referred by pediatric cardiologists. Regarding CHD severity, 637 (55%) had a defect of mild complexity. Of the patients analyzed, 616 (53%) had undergone an intervention, mainly atrial septal defect (ASD) closure, correction of tetralogy of Fallot, ventricular septal defect (VSD) closure and relief of coarctation of the aorta (CoAo). The main diagnosis of the 552 (47%) patients not submitted to an intervention were ASD, VSD, aortic stenosis, complex CHD and pulmonary stenosis. Regarding functional class, 1016 (87%) were in class I and 280 (24%) were lost to follow-up. Seventy-three patients had died, mainly due to cardiac death.

**Conclusion:**

In a unit were complex pediatric congenital heart surgery started twenty years ago, an increasing adult CHD workload was documented. Referral came predominantly from cities around the unit, most patients had low complexity defects and were in functional class I, a significant loss of follow-up was documented, and the death of patients was mainly due to the heart defect.

**Table:** 

Abbreviations, acronyms & symbols	
ASD	= Atrial septal defect
CHD	= Congenital heart disease
CoAo	= Coarctation of the aorta
ISACHD	= International Society for Adult Congenital Heart Disease
VSD	= Ventricular septal defect

## INTRODUCTION

The last decades have witnessed a considerable increase in the number of services dedicated to adults with congenital heart disease (CHD) ^[1,2]^. The ageing process of pediatric patients with simple defects as well as the increasing number of successful interventions for more complex cases are continuously demanding the creation of organized settings where these patients can be adequately assisted ^[3,4]^. Several aspects related to the information that can be obtained in the outpatient clinic require attention by the physicians in charge. The purpose of this paper is to describe our experience with adult CHD outpatients over the age of 16 attending a tertiary general university hospital in Brazil where surgery for complex neonatal CHD has been done for about 20 years. As far as we know, a full description of adult CHD patients’ characteristics followed in a specialized unit has not been reported and we believe this data gathering might be rewarding for other centers regarding their patients.

Setting: The University Hospital is a twelve-floor public general institution with 800 beds, founded in 1956 and attached to the Ribeirão Preto Medical School, São Paulo University. The recently opened Children’s Hospital is directly integrated to the main building, where adult patients are seen. The city has a current population of 720,000 people, but referral for tertiary treatment comes from a region of approximately 4 million inhabitants.

## METHODS

Since 2006, an Excel spreadsheet is being used routinely during the outpatient clinic. Weekly updated and backed up, it contains the basic information related to each patient. For the purpose of this investigation, we analyzed all data obtained until December 2017, annotating for each patient: age, gender, city of residence, main diagnosis, date and functional class at last examination, complexity of CHD^[5]^ and in-hospital referral pattern. When required, electronic and paper notes were searched. Acquisition of data regarding the deceased patients involved detailed analysis of patient’s notes, death certificate in some cases and telephone interview with a family member. If the information was not possible to obtain or was deemed unreliable, the cause of death was stated as unknown. Loss of follow-up was defined if the patient did not attend the clinic for at least once in the last two years.

## RESULTS

Until December 31, 2017, 1168 consecutive patients were registered at the clinic.

### Workload

**Fig. 1:**
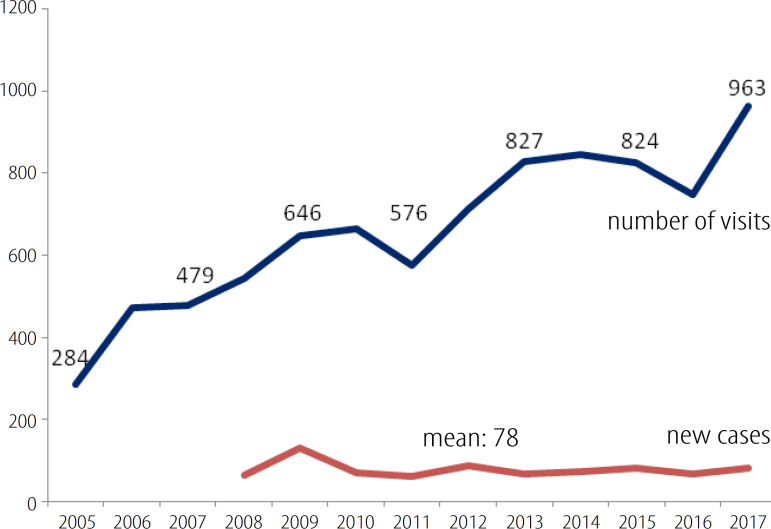
Number of outpatient visits and new cases during a 12-year period in an adult congenital heart disease unit.

The number of new cases/year has been stable since 2008 (mean 78), but an increasing number of visits to the clinic was verified, varying from 284 in 2005 to 963 in 2017 (Figure 1).

### Patient’s Residence

Of the patients referred, 920 (79%) live in the referral region, most of them no more than 300 km from the hospital, and 248 (21%) patients live in Ribeirão Preto city.

### In-Hospital Referral

**Fig. 2:**
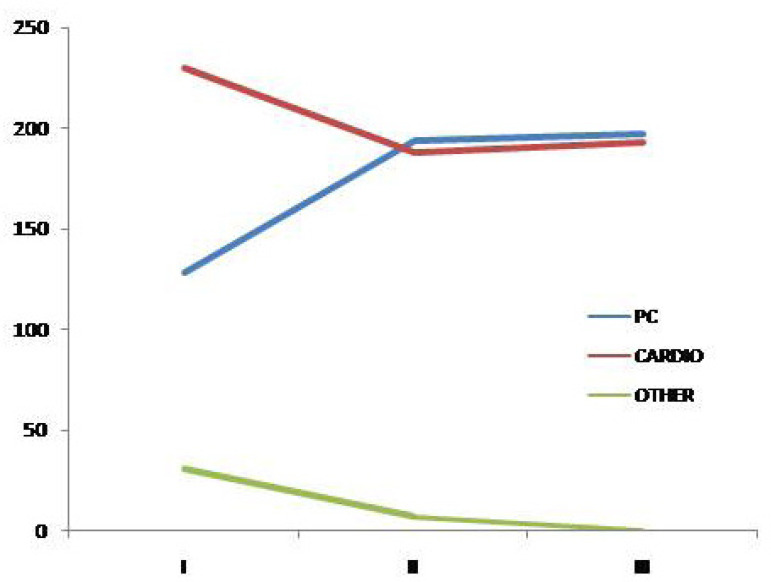
In-hospital referral pattern in 1168 patients during three periods equally divided based on the number of patients. I=patient number 1 to 389; II=patient number 390 to 779; III=patient number 780 to 1168. PC=pediatric cardiology clinic; CARDIO=general cardiology clinic

Referrals were made by hospital cardiologists for 611 (52%), while 519 (45%) were referred by pediatric cardiologists. A very small proportion of 30 (3%) patients reached the outpatient clinic by other means. Figure 2 shows this distribution as well as the referral tendency when patients are equally divided into 3 groups according to the number of patients.

### Patient’s Age

At the last examination, 663 (57%) patients were between 14 and 30 years old. Details are shown in Table 1.

**Table 1:** Age distribution in 1168 adults with CHD.

Age range	n	%
16-20	365	31
21-30	298	26
31-40	170	14
41-50	123	11
51-60	98	8
61-70	75	6
71-80	33	3
Over 80	6	1

### Heart Defect Complexity

Regarding severity, 637 (55%) patients had mild CHD, 437 (37%) had moderate CHD and 94 (8%) had severe CHD ^[5]^.

### Patients’ Characteristics

**Fig. 3:**
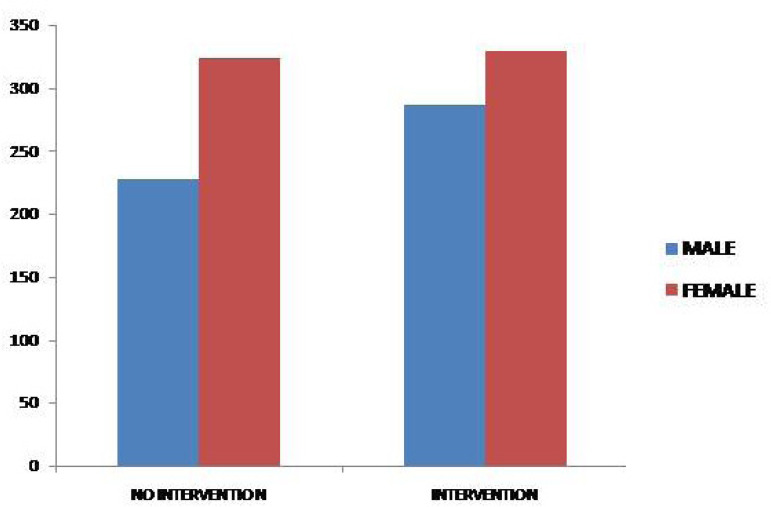
Number of individuals submitted or not to a therapeutic intervention according to gender in 1168 adult congenital heart disease patients.

Of the patients analyzed, 616 (53%) underwent percutaneous or surgical intervention. Among them, 329 (53%) were female and 287 (47%) were male. Among the 552 (47%) patients who were not treated invasively, 324 (59%) were female and 228 (41%) were male (Figure 3).

### Diagnosis 

**Fig. 4:**
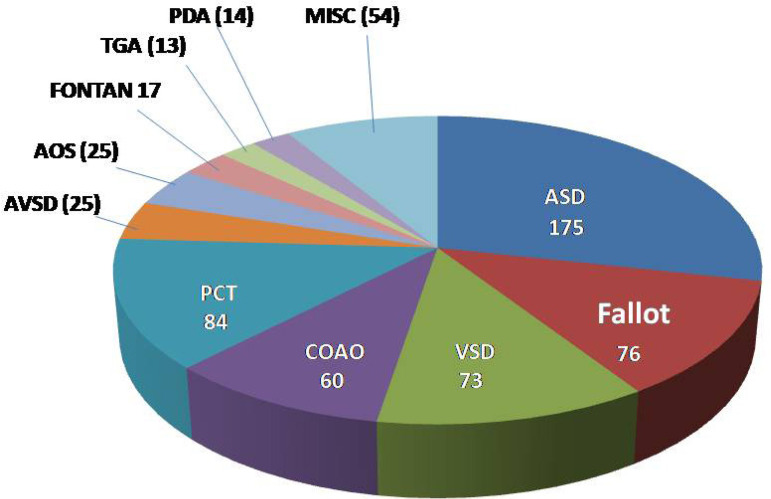
Diagnosis and number of patients undergoing intervention (n=616). ASD=atrial septal defect; AOS=aortic stenosis; AVSD=atrioventricular septal defect; CoAo=coarctation of the aorta; Fallot=tetralogy of Fallot; FONTAN=Fontan operation; MISC=miscellaneous; PCT=percutaneous intervention; PDA=ductus arteriosus; TGA=transposition of the great arteries; VSD=ventricular septal defect

Intervention: in 468 (76%) of the 616 patients submitted to an intervention, 175 had atrial septal defect (ASD) closure, 76 had correction of tetralogy of Fallot, 73 had ventricular septal defect (VSD) closure, 60 had surgical relief of coarctation of the aorta (CoAo) and 84 were submitted to a percutaneous intervention, mainly ASD occlusion, pulmonary valvoplasty and CoAo relief. Diagnosis details of these and other less frequent cases can be seen in Figure 4 and in Supplementary Table 1.

**Supplementary Table 1:** Diagnosis and number in descending order of 616 patients undergoing intervention.

Diagnosis	n
Atrial septal defect (n=175)	
Secundum	
Isolated	144
+ Pulmonary valve stenosis	10
+ Coronary artery disease	2
+ Mitral regurgitation	2
+ Mitral stenosis	1
+ Ductus arteriosus	1
Sinus venosus	
Isolated	7
Anomalous venous connection	8
Tetralogy of Fallot	76
Ventricular septal defect (n=73)	
Isolated	49
+ Pulmonary valve stenosis	9
+ Aortic regurgitation	7
+ ASD	5
+ Ductus arteriosus	1
+ Teratoma	1
+ Pulmonary valve stenosis + subaortic stenosis	1
Coarctation of the aorta	60
Percutaneous intervention (n=84)	
Pulmonary valvoplasty	30
ASD occlusion	24
CoAo relief	13
Ablation (WPW syndrome)	7
Ductus occlusion	5
Aortic valvoplasty	3
Coronary fistula	1
reCoAo relief	1
Atrioventricular septal defect (n=25)	
Partial	11
Complete	14
Left ventricular obstruction (n=25)	
Valvar	
Ross operation	5
Isolated	4
After CoAo relief	1
After VSD closure	1
Plus revascularization	1
Subvalvar	
Isolated	8
After VSD closure	1
Supravalvar	4
Fontan operation	17
Transposition of the great arteries (n=13)	
Senning	7
Jatene	4
Rastelli	2
Ductus arteriosus closure	14
Ebstein's anomaly of the tricuspid valve (n=6)	
Valve repair	4
Valve replacement	2
Total anomalous pulmonary venous connection	6
Mitral valve repair (n=6)	
Rheumatic	5
After correction of AVSD	1
Congenitally corrected TGA (n=5)	
ASD closure	3
Rastelli operation	1
Homograft replacement	1
Pacemaker implantation (n=5)	
Congenital AV block	4
CCTGA AV block	1
Glenn anastomosis	4
Double-outlet right ventricle (n=3)	
Correction	2
Blalock-Taussig anastomosis	1
Pulmonary valve atresia with VSD	2
ALCAPA	2
Pulmonary valve replacement	2
Cor triatriatum	2
Pulmonary artery banding (n=2)	
Univentricular heart	1
Partial left ventriculotomy	1
Switch back Ross operation	1
Coronary fistula ligation	1
Pericardiectomy	1
Left atrial myxoma	1
Dissecting aneurysm of the aorta	1
Scimitar syndrome	1
Pulmonary valve commissurotomy	1
Aortic arch interruption	1
Desmoid tumor after ASD closure	1

ALCAPA=anomalous origin of the left coronary artery from the pulmonary artery; ASD=atrial septal defect;AV=atrioventricular; AVSD=atrioventricular septal defect; CCTGA=congenitally corrected transposition of the great arteries; CoAo=coarctation of the aorta; TGA=transposition of the great arteries; VSD=ventricular septal defect; WPW=Wolf-Parkinson-White

**Fig. 5:**
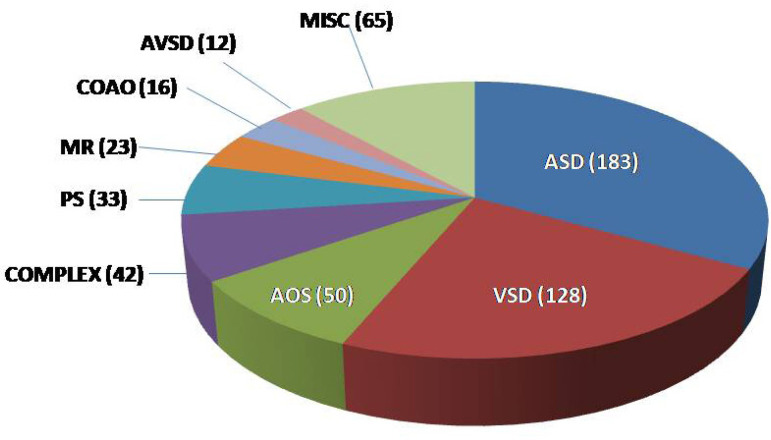
Diagnosis and number of patients not undergoing intervention (n=552). ASD=atrial septal defect; AOS=aortic stenosis; AVSD=atrioventricular septal defect; COMPLEX=complex congenital heart disease; CoAo=coarctation of the aorta; PS=pulmonary stenosis; MR=mitral regurgitation; MISC=miscellaneous; VSD=ventricular septal defect

No intervention: in 436 (79%) of the 552 patients not submitted to an intervention, the diagnoses were ASD (183), VSD (128), aortic stenosis (50), complex CHD (42) and pulmonary stenosis (33). Diagnosis details of these and other less frequent cases can be seen in Figure 5 and in Supplementary Table 2.

**Supplementary Table 2:** Diagnosis and number in descending order of 552 patients not undergoing intervention.

Atrial septal defect (n=183)	n
Secundum	
Isolated	
Small	51
Moderate-large	99
+ Pulmonary hypertension	4
+ Mild mitral regurgitation	1
+ Hypertrophic cardiomyopathy	1
+ Coronary artery disease	1
Patent foramen ovale	20
Sinus venosus	
Isolated	3
+ Anomalous pulmonary venous connection	2
+ Mild pulmonary stenosis	1
Ventricular septal defect (n=128)	
Small	
Isolated	113
+ Bicuspid aortic valve	6
+ Small ASD	2
+ Mild PS	2
+ Hypertrophic cardiomyopathy	2
+ Ductus arteriosus	1
+ Mild PS and subaortic stenosis	1
Large	1
Left ventricular obstruction (n=50)	
Valvar	
Mild	
Isolated	15
+ Ductus arteriosus	1
+ Mild mitral stenosis	1
Moderate-severe	11
Bicuspid aortic valve	8
Subvalvar	
Mild	9
Severe	1
Supravalvar	
Isolated	3
+ Branch PS	1
Complex congenital heart disease (n=42)	
Eisenmenger syndrome	
VSD	11
ASD	4
Truncus	3
Univentricular heart	2
Double-outlet right ventricle	1
Atrioventricular septal defect	1
Congenitally corrected transposition	1
Ductus arteriosus	1
Congenitally corrected transposition	
Isolated	3
+ Moderate-severe tricuspid regurgitation	6
+ Mild PS	2
+ Mild PS, VSD, ASD	1
+ Atrioventricular block	1
+ VSD and severe PS	1
Univentricular heart	
+ Mild PS	3
+ TGA and moderate PS	1
Right ventricular obstruction (n=33)	
Valvar	
Mild	
Isolated	20
+ Mild aortic valve stenosis	1
Moderate	4
Infundibular	
Isolated	3
+ Mild pulmonary regurgitation	1
Branch	4
Mild mitral regurgitation (n=23)	
Mitral valve prolapse	11
Congenital	6
Rheumatic fever	6
	
Coarctation of the aorta	16
Atrioventricular septal defect (n=12)	
Incomplete	10
Complete	2
Ebstein's anomaly of the tricuspid valve (n=10)	
Mild tricuspid regurgitation	
Isolated	7
+ Mild PS	1
Moderate TR	2
Small ductus arteriosus	8
Fistula (n=7)	
Coronary	5
Pulmonary arteriovenous	2
Marfan syndrome (n=6)	
Isolated	2
+ Dilated aorta	4
Arrhythmia (n=5)	
Supraventricular tachycardia	
WPW syndrome	1
Normal conduction system	1
Ventricular ectopy	1
Second-degree AV block	1
Congenital complete AV block	1
Tumor (n=4)	
Rabdomioma	2
Left ventricular fibroma	2
Pulmonary regurgitation (n=3)	
Mild	2
Severe	1
Dilated cardiomyopathy	4
Moderate tricuspid regurgitation	2
Mild aortic regurgitation	2
Rheumatic fever with no lesion	2
Uhl's anomaly	1
Takayasu syndrome with mild CoAo	1
Scimitar syndrome	1
Tetralogy of Fallot	1
Turner syndrome with dilated aorta	1
Cardiomyopathy in Duchenne syndrome	1
Kawasaki syndrome	1
Cor triatriatum with mild obstruction	1
Primary pulmonary hypertension	1
WPW syndrome	1
Ruptured sinus of Valsalva aneurysm	1
Partial pulmonary anomalous venous connection	1

ASD=atrial septal defect; AV=atrioventricular; CoAo=coarctation of the aorta; PR=pulmonary stenosis; TGA=transposition of the great arteries; TR=tricuspid regurgitation; VSD=ventricular septal defect; WPW=Wolf-Parkinson-White

Functional class: among 803 patients in active follow-up, 697 (87%) were in NYHA functional class I, 96 (12%) in class II and 10 (1%) in class III.

### Follow-Up

Regarding follow-up, 803 patients were in active follow-up, 9 were discharged, 30 were being followed elsewhere and 73 died; 253 (24%) patients were not seen in the last two years and were considered as lost to follow-up.

### Deceased Patients

Among the 73 deceased patients, 39 underwent a surgical procedure, while 34 did not. Thirty-two patients had a cardiac death, mainly due to heart failure. Twenty-three patients died of non-cardiac cause and in 18 cases the cause was unknown.

## DISCUSSION

It is well known that the adult CHD population exceeds the pediatric CHD population ^[6]^. This data should be considered a reward for many dedicated people and institutions in charge of children with CHD. Diagnostic improvement, proper intensive care, novel percutaneous interventions and nearly eight decades of congenital heart surgery have resulted in a progressively increasing number of individuals reaching adulthood, albeit frequently not cured ^[7]^. Most of them need a specialized setting to be followed where residual defects can be properly assessed, counseling be provided for many aspects of adult life and data are stored for multicenter studies, particularly regarding patients with moderate and severe complexity defects. Developed countries have responded well to this natural demand since the number of adult CHD units is increasing ^[2]^. Countries under a development process, however, feel the pressure. Health priorities and lack of an adequate infrastructure, particularly in densely populated areas, affect the creation of new adult CHD units, as well as resource improvement for those already established. Patients with moderate to high complexity CHD demand special attention. Many are survivors of a long-standing health problem with organic and psychological consequences that require understanding, counseling and appropriate action. Diagnostic and therapeutic resources necessary for an adequate patient management are expensive and data storage demands dedication, time and support from the institution.

The information provided here come from an institution where complex neonatal CHD surgery started to be offered routinely about twenty years ago. The adult CHD clinic, planned for care, data storage and training, was formally started 12 years ago. Before that, patients were being seen at general cardiology or pediatric cardiology clinics. Since then, all basic patient data are being updated weekly for helping patient management and also for scientific purposes ^[8,9]^. During this time, the outpatient workload analysis reveals two contrasting information: the increase in the number of visits and a stable number of new cases (Figure 1). More consultations are probably related to the ageing of the patient and also to the affluence of more complex cases, demanding more attention after leaving the pediatric surveillance. This increase in workload is well known ^[10]^. However, it should be noted that the exponential increase we verified in the 12-year period is somewhat influenced by a policy that does not conform to current guidelines. Until about two years ago, our patients were being seen at least once a year, including those with simple defects. Accepting the recommended policy of longer visit intervals for simple defects ^[3]^ and also realizing the need for more time to be devoted to complex cases resulted in a current two-to-three-year interval between clinical assessments for most cases of low complexity, always taking into consideration the individual situation. The stable number of new cases is intriguing. Three years ago, a letter was sent to all pediatricians and cardiologists in the region communicating the existence of a public adult CHD unit, with no effect on the number of new referrals so far.

The analysis of our patient’s residence adds to the well-known situation of adult CHD patients around the world, the great majority of them not being followed in a specialized setting. The prevalence of CHD in adulthood is 6.12 per 1000 individuals ^[11]^. This unique information was extrapolated and used to estimate the adult CHD population in different areas, despite regional differences may be detected. We found that only 248 (21%) of our patients live in the city which has a population of 720,000 inhabitants and should have, according to the above data ^[11]^ and based on the estimated population age distribution ^[12]^, approximately 3300 adults with some form of CHD. Referral to adult CHD patients is not compulsory to our hospital. Private care as well as secondary hospitals and clinics where many patients are probably being followed are widespread. Despite the estimated 92% patients not being followed in our unit is considered striking, the lack of adult CHD patients is a universal phenomenon and has been reported ^[1]^.

Most of our patients (52%) were referred from the general cardiology outpatient clinic. However, pediatric referral increased substantially in the last years due to a more frequent transfer which occurs at age 16 in our hospital (Figure 2). Transfer from pediatric to adult care should be rigorously done, as long as an adult CHD facility is offered. Keeping adults under pediatric surveillance is inadequate for patients and may mask the patient profile of a specialized unit.

The fact that 57% of our patients were under 30 years is related to the time of existence of the service. Complex CHD surgery started to be done about twenty years ago, and by 2006, many cases were still being followed in the pediatric clinic. This age range is somewhat different when compared to the patient’s age reported by pioneer institutions ^[13]^. This difference has some impact on the functional class and on the incidence of non-CHD problems.

Another important aspect to be observed in an adult CHD outpatient population is the complexity of the disease, which is fundamental for adequate patient care and follow-up policy. Despite the individual case has to be considered before complexity is defined, we are currently using the recently published guidelines ^[5]^. The finding that 637 (55%) of our patients were classified as patients with CHD of simple complexity demonstrates that our patient profile, as a whole, is made of not very complicated cases. This proportion is certainly different from other institutions experience. A recently published investigation from a pioneer center disclosed 52% of patients with simple CHD ^[14]^; however, their very complex cases were much more frequent (15%) when compared to ours (8%). Awareness that this outpatient profile will change over time is crucial. More complex cases will grow demanding more attention and resource allocation in the adult CHD clinic.

Regarding diagnosis, most (53%) of our patients underwent a percutaneous or surgical intervention. In nearly half of them (51%), the procedure was performed at or under 16 years of age. In 44% of patients, it occurred above the age of 18 and in 5% it was done at ages 17 and 18. Females (53%) predominated, which is possibly related to the large number of patients with ASD. All diagnosis can be seen in Supplementary Table 1. Surgical closure of an ASD was the most performed intervention, which is in agreement with most surgical series, particularly when the operation is done during adulthood ^[15]^. This practice has been changing in some centers where percutaneous occlusion is available ^[16]^. Some of our ASD cases were occluded percutaneously, taking into consideration the anatomic features, patient agreement and occluder availability. Correction of tetralogy of Fallot, closure of VSD and relief of CoAo were the other more frequent procedures performed in our patients. A percutaneous intervention in our setting is usually performed for ASD occlusion and relief of pulmonary valve stenosis and CoAo.

Looking at the 47% of our patients who had not undergone an intervention, we can see that the great majority of them had simple CHD, like ASD and VSD, and a few had a complex defect. It should be emphasized that some of these patients were in the waiting list for a procedure at the time of this analysis. The entire diagnosis can be seen in Supplementary Table 2.

Using the NYHA criteria, we found that among the 803 patients under routine follow-up, 697 (87%) were in functional class I, 96 (12%) in class II and 10 (1%) in class III. In a traditional British institution, where severe complexity CHD is more frequent, these numbers were 65%, 28% and 7%, respectively ^[13]^.

Follow-up is essential in any medical area to protect patients and to know the therapeutic results. The information obtained might help in the management and, ideally, should be used for multicentric studies regarding several aspects of this patient population ^[17]^. As cure is rare, most adult CHD patients should have lifelong follow-up ^[18]^, including those with simple defects ^[19]^, since complications may affect survival and quality of life ^[20]^. Adult CHD loss of follow-up has been previously documented and some risk factors have been already determined ^[21]^. Our finding of 24% of patients not seen in the last two years is a matter of concern. The results of an active search program at our institution revealed that 47% of the patients who returned to the clinic stated they did not know about the need for follow-up and that 52% of them considered themselves cured, even some with non-simple CHD. The analysis of these unpublished observations allowed us to conclude that education was probably not adequately provided for most of these patients. Patient education is crucial ^[22]^ and should start in the pediatric age.

Mortality in adult CHD patients is a crucial matter. A recent report from Australia disclosed 11% of deaths among their cases ^[23]^ and in a related editorial ^[24]^, reviewing several centers experience, this number was found to vary between 3.3% to 16%. Our 8% (73 cases) mortality is lower than the Australian number mentioned above, probably because the complexity of their cases was more severe than ours (52% to 36% of moderate-severe complexity). Most of the deaths in their experience were non-cardiac (54%) while in our cases 32 (58%) were cardiac. Heterogeneous groups of patients with different cardiac defects may explain the wide difference between reported services ^[24]^. It should be mentioned that the predominant cardiac mortality in our experience is different from the more traditional institutions where a clear shift from perioperative to chronic cardiac mortality and to non-cardiac death was verified ^[14]^. As well stated ^[24]^, defining the circumstances of death can be difficult and, in our particular case, due to a high (23%) incidence of patients with cause of death stated as unknown, a current investigation specifically devoted to patient death is under way.

## CONCLUSION

The demands of an adult CHD outpatient clinic are enormous, if an adequate assistance is to be provided. Awareness of simple details such as the age and residence of the patients as well as the clinic workload pattern should stimulate the search for those who are not under specialized surveillance. A proactive attitude of the physician is expected. Diagnostic accuracy with proper definition of patient complexity is crucial to establishing the consultation interval. Adequate follow-up is essential and should be a special target. However, achieving a perfect model of care is not easy. Human and structural resources are needed, and, in our particular case, we are far from ideal. As it has been wisely said some time ago, the provision of healthcare services for patients with CHD should be reshaped, since most of them are adults. Medical and non-medical education regarding the needs of these patients should be implemented. Published guidelines are useful and should be followed. Adult CHD is a universal matter and The International Society for Adult Congenital Heart Disease (ISACHD) is playing a key role in coordinating the future advances in the care of adults with CHD worldwide. In our view, every physician in charge of an adult CHD clinic should strive to finding lost patients, provide evidence-based treatment and establish a good follow-up program.

**Table:** 

Author's roles & responsibilities	
FTVA	Substantial contributions to the conception or design of the work; or the acquisition, analysis, or interpretation of data for the work; final approval of the version to be published
PHM	Acquisition, analysis, or interpretation of data for the work; final approval of the version to be published
MFBJ	Acquisition, analysis, or interpretation of data for the work; final approval of the version to be published
AS	Acquisition, analysis, or interpretation of data for the work; final approval of the version to be published
